# A Mobile Phone–Based Approach for Hearing Screening of School-Age Children: Cross-Sectional Validation Study

**DOI:** 10.2196/12033

**Published:** 2019-04-01

**Authors:** Yuan-Chia Chu, Yen-Fu Cheng, Ying-Hui Lai, Yu Tsao, Tzong-Yang Tu, Shuenn Tsong Young, Tzer-Shyong Chen, Yu-Fang Chung, Feipei Lai, Wen-Huei Liao

**Affiliations:** 1 Graduate Institute of Biomedical Electronics & Bioinformatics National Taiwan University Taipei Taiwan; 2 Information Management Office Taipei Veterans General Hospital Taipei City Taiwan; 3 Big Data Center Taipei Veterans General Hospital Taipei City Taiwan; 4 Department of Otolaryngology-Head and Neck Surgery Taipei Veterans General Hospital Taipei Taiwan; 5 Department of Medical Research Taipei Veterans General Hospital Taipei Taiwan; 6 Department of Otolaryngology-Head and Neck Surgery School of Medicine National Yang-Ming University Taipei Taiwan; 7 Department of Speech Language Pathology and Audiology National Taipei University of Nursing and Health Sciences Taipei Taiwan; 8 Department of Biomedical Engineering National Yang-Ming University Taipei Taiwan; 9 Research Center for Information Technology Innovation Academia Sinica Taipei Taiwan; 10 Holistic Education Center Mackay Medical College Taipei Taiwan; 11 Department of Information Management Tunghai University Taipei Taiwan; 12 Department of Electrical Engineering Tunghai University Taipei Taiwan; 13 Department of Computer Science & Information Engineering National Taiwan University Taipei Taiwan; 14 Department of Electrical Engineering National Taiwan University Taipei Taiwan

**Keywords:** hearing tests, telemedicine, mobile apps, audiometry, pure-tone

## Abstract

**Background:**

Pure-tone screening (PTS) is considered as the gold standard for hearing screening programs in school-age children. Mobile devices, such as mobile phones, have the potential for audiometric testing.

**Objective:**

This study aimed to demonstrate a new approach to rapidly screen hearing status and provide stratified test values, using a smartphone-based hearing screening app, for each screened ear of school-age children.

**Method:**

This was a prospective cohort study design. The proposed smartphone-based screening method and a standard sound-treated booth with PTS were used to assess 85 school-age children (170 ears). Sound-treated PTS involved applying 4 test tones to each tested ear: 500 Hz at 25 dB and 1000 Hz, 2000 Hz, and 4000 Hz at 20 dB. The results were classified as *pass* (normal hearing in the ear) or *fail* (possible hearing impairment). The proposed smartphone-based screening employs 20 stratified hearing scales. Thresholds were compared with those of pure-tone average (PTA).

**Results:**

A total of 85 subjects (170 ears), including 38 males and 47 females, aged between 11 and 12 years with a mean (SD) of 11 (0.5) years, participated in the trial. Both screening methods produced comparable *pass* and *fail* results (pass in 168 ears and fail in 2 ears). The smartphone-based screening detected moderate or worse hearing loss (average PTA>25 dB) accurately. Both the sensitivity and specificity of the smartphone-based screening method were calculated at 100%.

**Conclusions:**

The results of the proposed smartphone-based self-hearing test demonstrated high concordance with conventional PTS in a sound-treated booth. Our results suggested the potential use of the proposed smartphone-based hearing screening in a school-age population.

## Introduction

### Background

Worldwide, more than 466 million (over 5%) people, including 34 million children, are estimated to have a hearing impairment. Hearing impairment is difficult to monitor because of the limited availability of testing equipment and trained specialists in many developing countries [1,2]. Unidentified hearing impairment has been one of the most common disorders in school-age children [1,3,4]. Several studies have shown that children with hearing impairments remain unidentified, and if they do not receive treatment, these children may experience a delay in the acquisition of speech and language skills [5-7]. The burden of hearing loss is the greatest in developing countries and more than 80% of people with hearing loss live in these areas [3,8]. However, hearing care services in these areas are either very limited or absent altogether [8,9]. Early detection and early intervention are key factors in reducing the impact of hearing impairment on the development and future achievement in school-age children [10].

Pure-tone screening (PTS) is considered as the gold standard for hearing screening programs for school-age children [11,12]. PTS is usually administered by a hearing professional or a nurse, using a portable instrument that produces a limited set of test stimuli often at a predetermined level between 20 and 40 dB hearing level (HL), depending on the age of the group being tested [2]. Current school-based hearing screening protocols have not been standardized, and numerous screening criteria vary according to the guidelines of the agency, state, or country. For example, the American Speech-Language-Hearing Association (ASHA) and the American Academy of Audiology published professional recommendations that specify screening at 20 dB at frequencies of 1000 Hz, 2000 Hz, and 4000 Hz [2,4]. In 2003, the American Academy of Pediatrics (AAP) also suggested screening at 20 dB at frequencies of 500 Hz, 1000 Hz, 2000 Hz, and 4000 Hz [3]. One major drawback of the current hearing screening methods is the lack of sensitivity and specificity in determining hearing ability and indicating hearing loss candidacy. As a result, conventional PTS provides only a pass or fail result for each screened ear and lacks hearing status assessment and further stratified test values as provided by tools such as the Landolt C eye chart for follow-ups [6,7].

The Hearing Scale Test (HST) is a novel hearing screening method derived from the consecutive hearing screening procedures for approaching the current hearing status of each screened ear of children [5,8]. The HST employs stratified hearing scales containing 4 test tones (500 Hz, 1000 Hz, 2000 Hz, and 4000 Hz), where adjacent scales differ from each other by 5 dB (Table 1). In addition to the pass/fail results that most PTS-based screening programs offer, the HST also offers current hearing status and provides stratified test values that can be recorded for follow-ups. Our previous studies have shown that the automated audiometry devices based on personal computers built with the hearing protocol of the HST, which offer a user-friendly interface and measure hearing threshold values, are useful for monitoring progressive hearing changes in school-age children [5,8].

Automated audiometry devices have demonstrated that comparable hearing threshold values, compared with those obtained by automated audiometry, such as computer-assisted audiometry [9,10,12] or smartphone-based audiometry [11,13-20], and results obtained by audiologists using conventional manual audiometry can be achieved. Automated audiometry devices using mobile phone require the use of earphones, and given the huge variety of combinations of earphones and mobile phone, standardized and calibrated software and devices continue to be the key for performing reliable hearing tests [15,16,21-27]. Apple, iOS-based devices provide standardized hardware and software components; therefore, most apps can potentially be universally shared with all iOS-based device models [19]. Numerous audiometric apps have been developed for hearing assessments on Apple mobile devices [19,21,28], most of which calibrate mobile devices using a biological method to determine a reference sound level in relation to the hearing threshold of normal people [11,15,22]. To avoid possible variability and inconsistency caused by biological calibration, our previous study has shown that reference equivalent threshold sound pressure levels (RETSPLs) represent a reliable calibration method for output levels across different Apple mobile devices with bundled earphones [23].

### Objectives

In this study, we developed an iOS-based smartphone hearing test app *Ear Scale* and evaluated its performance and feasibility as a hearing screening program for school-age children. We investigated the accuracy of the hearing tests conducted on mobile devices calibrated by RETSPLs for Apple EarPod [23]. We compared the performance of the smartphone-based automated hearing screening with that of audiologist-assisted pure-tone audiometry (PTA) performed in a sound-treated booth. Different screening protocols, including those suggested by the AAP and ASHA, were also compared with the built-in HST protocol of the Ear Scale app [15,16,19,21-27,29].

**Table 1:** Stimulus levels in dB hearing level for tested frequencies in the proposed Hearing Scale Test.

Stimulation level		Hearing Scale Test									
		Normal (pure-tone audiometry ≤25 dB)					Possible hearing impairment (pure-tone audiometry >25 dB)				
		S^a^_1_	S_2_	S_3_	S_4_	S_5_	S_6_	S_7_	S_8_	S_9_	S_10_
**Frequency (Hz)**											
	1000 Hz, 2000 Hz, and 4000 Hz	0	5	10	15	20	25	30	35	40	45
	500 Hz	5	10	15	20	25	30	35	40	45	50

^a^S: stratified hearing scale.

## Methods

### Study Setting and Participants

This prospective cohort study was conducted at an elementary school in Taipei, Taiwan. We recruited children from grades 5 and 6, aged between 11 and 12 years. A total of 85 children (38 boys and 47 girls) were enrolled, with 170 ears tested. The trial was approved by the Institutional Review Board of Taipei Veterans General Hospital (2017-10-003CC). Written informed consent was collected by the teachers from the parents, before the scheduled date of the hearing screening tests. After instruction by the researchers, each child, in a random order, underwent smartphone-based and booth-based hearing screening consecutively. The smartphone-based hearing screening procedures were performed in a quiet room in the school. Before the hearing screening, the students were taught how to wear the headphones and push a button when hearing the tone. The air conditioner was turned off during the measurements to reduce ambient noise, the level of which was monitored every 30 min by a sound level meter to ensure an ambient noise level of less than 50 dB at test frequencies of 500 Hz, 1000 Hz, 2000 Hz, and 4000 Hz.

### Measurements

#### Pure-Tone Screening Procedures in a Sound-Treated Booth

The audiologist manually controlled a GrasonStadler GSI 18 screening audiometer that was used with a Telephonics TDH-39 supraaural earphones previously calibrated according to International Organization for Standardization (ISO) 389-1. A *pass* result for an ear indicated that the child responded correctly to all 4 test tones. If the child did not respond to all 4 test tones after 2 consecutive testing procedures, then the ear was assigned a *fail* result. PTA hearing thresholds of more than 25 dB at 500 Hz, 1000 Hz, 2000 Hz, and 4000 Hz in the sound-treated booth were designated as *hearing impairment*.

#### iOS Automated Audiometry App

**Figure 1:**
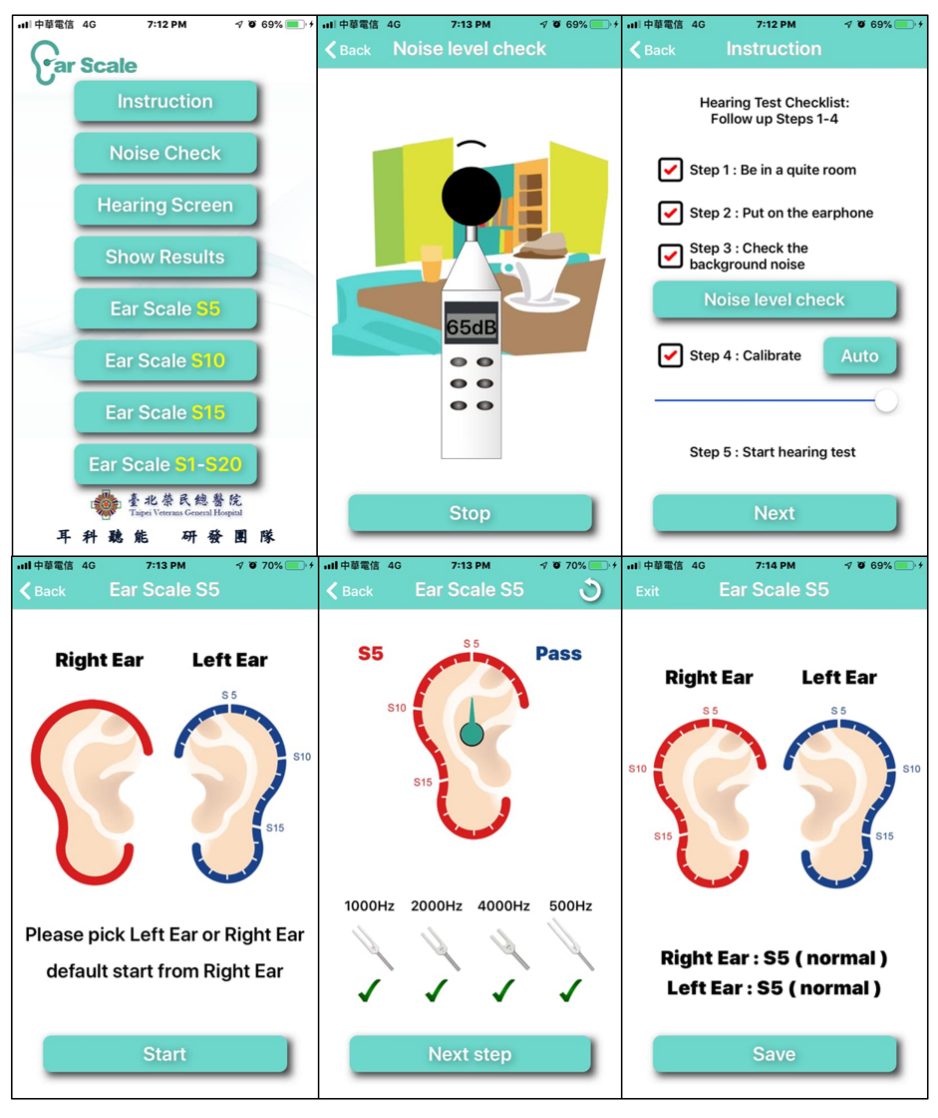
Screenshot of the Ear Scale app includes instructions for the testers and the hearing test process.

**Figure 2:**
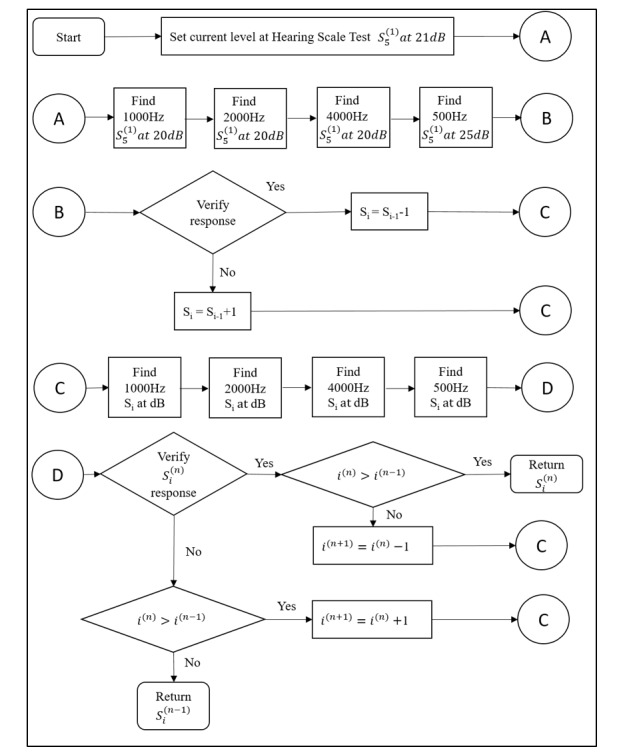
The computerized smartphone-based hearing screening flow diagram. S: stratified hearing scale.

The iOS-based automated *Ear Scale* app (version 2.0) was developed to perform pure-tone air conduction hearing testing and was made freely accessible as a download through the Apple iTunes store in 2018. The HST, a new modified hearing screening method derived from consecutive hearing screening procedures to assess the current hearing status of each screened ear of children, was used to determine the hearing threshold [5] of each screened ear in children (Table 1). The test tones were 1.5 seconds in duration, whereas the silent interval between successive tones randomly varied between 2 and 3 seconds, and depending on the user response, the sound intensity was changed in steps of 5 dB semiautomatically [19]. The test tone’s amplitude was modulated with a depth of 100% [11]. At the end of the test, an audiogram was displayed, which could be saved on the device (Figure 1). The Ear Scale app involved computerized self-determination of the lowest audible sound generated by the mobile device. The computerized smartphone-based audiometer presented the 4 test tones of the HST at the appropriate stimulus levels semiautomatically, as shown in Figure 2. The Ear Scale app started with a hearing scale of 25 dB (S_5_; Figure 2). The 4 test tones were automatically presented in a fixed order: 1000 Hz, 2000 Hz, 4000 Hz, and 500 Hz. If the child responded correctly to all test tones of a particular hearing scale, then the test stimulus level was decreased (corresponding to hearing scales decreasing from S_4_ to S_1_) until the child did not respond to any of the 4 test tones; otherwise, the test stimulus level was increased (corresponding to hearing scales increasing from S_6_ to S_10_; Figure 2). The minimum audible hearing scale on the HST indicated the stimulus level at which the child responded correctly to all 4 test tones. If the child did not respond correctly to hearing scale S_10_, then the result was designated as *no response* (NR). Scales S_1_ to S_5_ of the HST are equivalent to a PTS *pass* result, whereas scales S_6_ to S_10_ and NR are equivalent to a PTS *fail* result (Figure 2). The tests on mobile devices were conducted twice, test and retest.

#### iOS Automated Audiometry Calibration

Calibration of iOS-based devices with Apple EarPod RETSPLs was described in detail in a previous paper [23]. Briefly, the RETSPL method of the hearing self-test carried out on mobile devices with calibrated bundled headphones is used when calibrating audiometric equipment to a hearing threshold of 0 dB at various frequencies. Pure-tone stimuli at 250 Hz, 500 Hz, 1000 Hz, 2000 Hz, 4000 Hz, and 8000 Hz were generated on the iOS mobile device and delivered by the Apple EarPods. The KEMAR manikin was developed to meet the needs of hearing aid designers and other manikin users. The EarPods were placed in the left and right pinna of the KEMAR manikin for eardrum-pressure recording. Hearing thresholds were determined by the ascending method described in ISO 8253-1 [24], where the step size was set to 1 dB. The initial level was set at 10 dB below the lowest subject response level, which was predetermined using a conventional audiometer. Subjects were instructed to respond when they heard the stimulus. Final thresholds were determined using a 2-down, 1-up adaptive staircase procedure [25] after 3 reversals. All devices were standardized by setting the user-controllable volume to 100% of its maximum limit. The maximum difference between right and left EarPods was less than 1 dB and the maximum difference among devices (iPhone 5s, iPhone 6, iPhone 6 Plus, iPhone 7, iPhone 7 Plus, and iPad mini) was less than 1.5 dB with output levels across 5 EarPods between 250 and 8000 Hz on a single device (iPad mini 4). The maximum difference was less than 1.0 dB. The microphone of the ear simulators and the electrical and acoustical measurement systems were calibrated using a GRAS model 42AA pistonphone. The output levels of the EarPods at 500 Hz, 1000 Hz, 2000 Hz, and 4000 Hz were calibrated in units of dB sound pressure level (SPL) when the volume of the Apple mobile device was set to maximum. The output level (dB) of the pure-tone sound corresponding to each hearing test frequency is similar to that of the apparatus previously described for sound output calibration [19,23]. Apple EarPod RETSPLs have stable output levels between right and left EarPods, which can be applied to calibrate output levels of various Apple mobile devices with EarPods [23].

### Statistical Analysis

**Figure 3:**
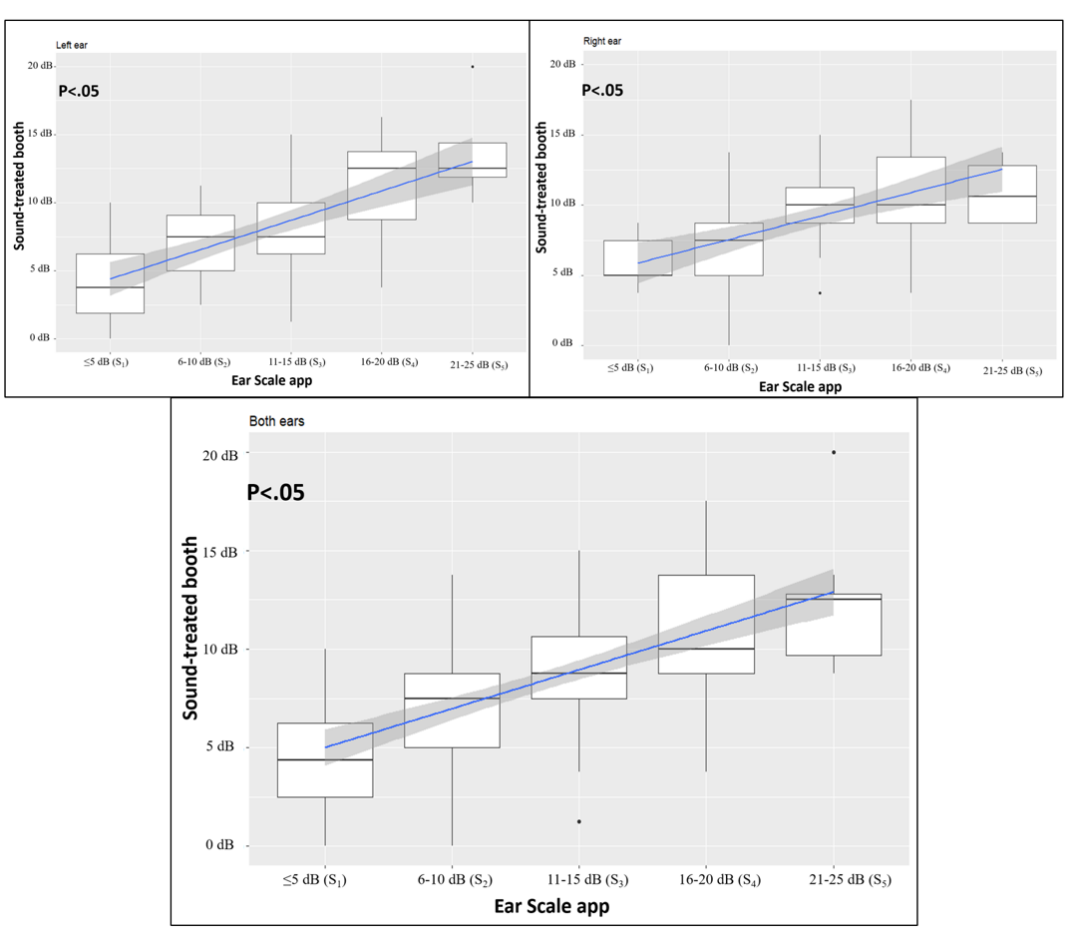
Box plots of the hearing results of right ears and left ears obtained from the Ear Scale app in relation to those obtained from pure-tone screening. The box includes the median (heavy line) and represents the first and third quartiles, whereas the vertical bar indicates the SD. Blue lines represent best-fit linear regressions of the means of the boxes, whereas the gray areas around the line represent the 95% CI of the model (P<.05, differences were found between groups). S: stratified hearing scale.

For hearing screening, the presence or absence of hearing loss (PTA>25 dB) in each ear was determined by sound-treated booth audiometry. The results from the Ear Scale app were compared with the threshold obtained from sound-treated booth PTA measurement. These data were entered into 2×2 tables to calculate the sensitivity, specificity, positive predictive value, and negative predictive value. The hearing scale obtained from the Ear Scale app and the corresponding mean pure-tone threshold obtained from the sound-treated booth are shown by a box plot (Figure 2). The corresponding pure-tone threshold of each grade of the HST is shown by a box plot (Figure 3). The correlation coefficient was calculated to estimate the average correlation coefficient across both methods. The Kruskal-Wallis test was performed to determine significance. Analyses were performed using the SPSS version 23.0 (SPSS Inc) and Microsoft Excel version 2016 (Microsoft Inc) for personal computers. *P* values less than .05 were considered statistically significant. The PTA thresholds at 500 Hz, 1000 Hz, 2000 Hz, and 4000 Hz were summarized as the mean (SD) values (Table 1).

## Results

### Comparing 2 Hearing Screening Methods: Conventional Pure-Tone Screening Versus the Ear Scale App

Of the 170 ears tested by sound-treated booth PTA, 98.8% (168/170) and 1.2% (2/170) were assigned *pass* and *fail* results, respectively. Similarly, of the 170 ears tested by the Ear Scale app, 98.8% (168/170) and 1.2% (2/170) of the tests were assigned *pass* and *fail* results, respectively (Table 2). The results using these 2 methods of hearing screening were calculated in a 2×2 table to determine the sensitivity, specificity, positive predictive value, and negative predictive value (Figure 3). In addition to the dichotomous *pass* or *fail* results, the Ear Scale app provided stratified hearing scales for each screened ear. The results of 84 left ears with a *pass* result were stratified as 0 dB (S_1_) of 13% (11/84), 5 dB (S_2_) of 38% (32/85), 15 dB (S_3_) of 33% (28/85), 20 dB (S_4_) of 11% (9/85), and 25 dB (S_5_) of 4% (4/85), whereas *fail* results were stratified as 35 dB (S_7_) of 1% (1/85). Similarly, 84 *pass* results and 1 *fail* result for right ears were also further stratified. The results of 168 *pass* ears and 2 *fail* ears are pooled and shown in Table 3.

**Table 2:** Participants’ demographics and hearing impairment candidacy (as graded by the pure-tone screening and Hearing Scale Test).

Variables		Statistics
Participants, n		85
Age (years), mean (SD)		11 (0.5)
**Gender, n**		
	Male	38
	Female	47
**Pure-tone screening, n**		
	≤25 dB (normal)	168
	26-40 dB (mild loss)	2
	41-55 dB (moderate loss)	0
	56-70 dB (moderate to severe loss)	0
	71-90 dB (severe loss)	0
	≥91 dB (profound loss)	0
**Ear Scale app with the Hearing Scale Test, n**		
	≤ 25 dB (S^a^_1_-S_5_, normal)	168
	>25 dB (S_6_-S_10_, hearing loss candidate)	2

^a^S: stratified hearing scale.

### Validation of the Built-In Hearing Scale Test Hearing Screening Protocol for the Ear Scale App

As the HST was used in our Ear Scale app for the default screening protocol, we also compared the HST with other popular protocols, including those suggested by the AAP and ASHA. The Ear Scale app was highly accurate at the tested frequencies (500 Hz, 1000 Hz, 2000 Hz, and 4000 Hz) for all 3 screening protocols. The specificity was 100% and the sensitivity was 100% for HST (1000 Hz, 2000 Hz, and 4000 Hz at 20 dB and 500 Hz at 25 dB), 95.2% for AAP (500 Hz, 1000 Hz, 2000 Hz, and 4000 Hz at 20 dB), and 95.2% for ASHA (500 Hz, 1000 Hz, 2000 Hz, and 4000 Hz at 15 dB). The false-positive rate was 0% in all 3 screening protocols, whereas the false-negative rates were 0% of HST, 4.8% of AAP, and 4.8% of ASHA, respectively. A summary of the results from all 3 tested screening protocols is provided in Table 4.

### Accuracy of Ear Scale App Calibration at All Hearing Scale Test Grades

The correlation between the 2 measurements by utilizing the Ear Scale app in a quiet conference room and the clinical audiometer in a sound-treated room was significant at the .01 level (Figure 3). Statistically significant differences were found in all tested HST scales (S_1_, S_2_, S_3_, S_4_, and S_5_) in right ears and left ears (Kruskal-Wallis test with 5 degrees of *P*<.01; Figure 3). Similarly, the pooled data from both ears also showed a significant difference, indicating the usefulness of the proposed Ear Scale app in not only distinguishing ears with *pass* or *fail* results but also providing an accurate measurement of the HL of school children.

**Table 3:** The Hearing Scale Test and the mean difference between thresholds (dB) for the Ear Scale app and sound-treated booth (N=170 ears).

Ear Scale app with Hearing Scale Test		Sound-treated booth in pure-tone screening	
		Mean (SD)	n
**Left ear (mean thresholds)**			
	≤5 dB (S^a^_1_)	4 (3.14)	11
	6-10 dB (S_2_)	7 (2.7)	32
	11-15 dB (S_3_)	8 (2.9)	28
	16-20 dB (S_4_)	11 (4.2)	9
	21-25 dB (S_5_)	14 (4.3)	4
	26-30 dB (S_6_)	0	0
	31-35 dB (S_7_)	31 (NaN^b^)	1
	36-40 dB (S_8_)	0	0
	41-45 dB (S_9_)	0	0
	46-50 dB (S_10_)	0	0
**Right ear (mean thresholds)**			
	≤5 dB (S_1_)	6 (2.1)	5
	6-10 dB (S_2_)	7 (3.3)	26
	11-15 dB (S_3_)	10 (2.6)	31
	16-20 dB (S_4_)	11 (3.8)	18
	21-25 dB (S_5_)	11 (2.6)	4
	26-30 dB (S_6_)	0	0
	31-35 dB (S_7_)	0	0
	36-40 dB (S_8_)	36 (NaN)	1
	41-45 dB (S_9_)	0	0
	46-50 dB (S_10_)	0	0
**Both ears (mean thresholds)**			
	≤5 dB (S_1_)	5 (2.9)	16
	6-10 dB (S_2_)	7 (3.0)	58
	11-15 dB (S_3_)	9 (2.8)	59
	16-20 dB (S_4_)	11 (3.8)	27
	21-25 dB (S_5_)	12 3.6)	8
	26-30 dB (S_6_)	0	0
	31-35 dB (S_7_)	31 (NaN)	1
	36-40 dB (S_8_)	36 (NaN)	1
	41-45 dB (S_9_)	0	0
	46-50 dB (S_10_)	0	0

^a^S: stratified hearing scale.

^b^NaN: not a number.

**Table 4:** Comparison of the hearing screening protocols for both ears of all subjects participating in the study.

Results	Hearing screening protocols		
	Hearing Scale Test, %	American Academy of Pediatrics, %	American Speech-Language-Hearing Association, %
Sensitivity	100	95.2	95.2
Specificity	100	100	100
False-positive	0	0	0
False-negative	0	4.8	4.8

## Discussion

### Principal Findings

The findings from this study support the use of the Ear Scale app in smartphone-based hearing screening of school children. To the best of our knowledge, this is the first report proposing a method for stratifying hearing test results on a smartphone and then using it for hearing screening in school children. As hearing screening is useful for detecting hearing impairment in the school system [26], we developed the Ear Scale app to evaluate school children’s HL ranges on the basis of 20 stratified hearing scales, that is, 5 dB (S_1_) to 100 dB (S_20_), plus an NR result. Our Ear Scale 25 dB (S_5_) menu item fit a normal hearing range, the Ear Scale 50 dB (S_10_) menu item fit a mild hearing loss range, the Ear Scale 75 dB (S_15_) menu item fit a moderate hearing loss range, and the Ear Scale app with the HST from 5 dB (S_1_) to 100 dB (S_20_) menu item can be customized for a wide range of hearing loss for school-age children. Conventional PTS provides a *pass* / *fail* result, and it therefore provides little information regarding a child’s hearing ability. The Ear Scale app with the HST proposed in this study has 10 stratified hearing scales from 0 dB (S_1_) to 45 dB (S_10_) plus an NR result. The Ear Scale app with the HST is derived from the hearing screening concept of dichotomized test results (*pass* or *fail*), but the use of computerized hearing screening procedures and hearing scales with different test stimulus levels allows the minimum audible hearing scale to be determined. The scale determined by the Ear Scale app can present the current hearing status of each tested ear. The Ear Scale app with the HST can rapidly evaluate the hearing status of the tested ear, typically within 3 to 5 min.

Many different ear screening protocols have been established in the past [7,30], but the methods suitable for children and school-age groups have not been standardized [27,30]. The Ear Scale app described in this study has several implications for hearing screening programs. First, the built-in HST protocol stratifies the hearing scales of each screened ear, whereas PTS provides only *pass* or *fail* results (Table 2). These stratified hearing scales from 0 dB (S_1_) to 45 dB (S_10_) recorded in an initial hearing assessment can be used for further follow-up surveillance in hearing screening programs [5,8]. Second, the results of the HST show the distribution of different stratified hearing scales (representing different degrees of hearing status) of all screened ears with the same median reference standard (S_5_), thus facilitating comparisons of hearing screening results among classes or schools (Table 3). The Ear Scale app with a computerized audiometer typically requires only 3 to 5 min per child, whereas PTS conducted manually requires 1 to 2 min per child. The longer testing time of the Ear Scale app is because of the stratification performed by consecutive tests to determine the minimum audible hearing scale. However, this small increase in the time spent in the test is worthwhile to achieve the goal of determining a more informative hearing status associated with the use of stratified hearing scales in the Ear Scale app.

It is projected that the smartphone subscription will increase from 5 billion in 2018 to 7.2 billion in 2024 [29], and there has been a surge of health-related smartphone apps in recent years [31-36]. Smartphone hearing screening audiometry has been widely implemented as mobile phone gained popularity, and several studies have compared hearing thresholds with standardized automated hearing thresholds obtained in a sound-treated booth [11,13,14,18,28,37-39]. However, none of these studies integrated a computerized hearing screening flow diagram with a graphical interface for school-age children. Our Ear Scale app is based on a series of distinct steps and is implemented in the form of an automated process, which improves standardization of the test procedures and therefore avoids inconsistency [40,41].

Our results indicate that the iOS-based Ear Scale app is reasonably accurate for hearing screening. The sensitivity and specificity were high (100%), whereas the false-positive (0%) and false-negative rates (0%) were low when the hearing tests were performed in a quiet room in the school library, ensuring an ideal test for hearing screening. The Ear Scale app was also found to be highly accurate in testing several hearing screening protocols in addition to the built-in HST [5], including those recommended by the AAP [3] and ASHA [2]. The Ear Scale app can be used to screen school-age children and individuals at a high risk of developing hearing loss and facilitate early detection of abnormal or worsening thresholds. The Ear Scale app is therefore an appropriate tool to screen for disabling hearing loss and detect hearing loss in a nonsoundproof environment. Children who have limited access to audiologists may benefit from a smartphone-based, freely available self-assessment hearing screening test such as this. With increasing rates of age- and noise-related hearing loss globally, further studies are required to examine the suitability of the Ear Scale app for early detection or prevention of hearing loss in the future.

### Limitations

The environmental noise level is one of the most common concerns in hearing screening [7,11,27,30,42,43]. This study was conducted at a school, where ambient noise levels were increased but not excessive at various times, which may have influenced the findings. Therefore, recalibration is required to reset RETSPLs and maximum output levels with bundled earphones (Apple EarPods) for each new device model. At the same time, we must recalibrate the mobile devices with the KEMAR manikin, following the same procedures to obtain the mean values [44].

### Conclusion

This paper proposes an innovative approach to hearing screening of school-age children. We developed an Ear Scale app that is comparable with clinical-grade PTS in a sound-treated booth in terms of hearing test results. With favorable high sensitivity and specificity rates and low false-positive and false-negative rates, this study demonstrated that using the proposed Ear Scale app can rapidly screen hearing status and provide stratified test values for each screened ear, and it is therefore an ideal tool for hearing screening in schools.

## Abbreviations

AAP: American Academy of Pediatrics
ASHA: American Speech-Language-Hearing Association
HL: hearing level
HST: Hearing Scale Test
ISO: International Organization for Standardization
NR: no response
PTA: pure-tone average
PTS: pure-tone screening
RETSPL: reference equivalent threshold sound pressure level
S: stratified hearing scale
SPL: sound pressure level

## References

[1] Berg AL, Papri H, Ferdous S, Khan NZ, Durkin MS (2006). Screening methods for childhood hearing impairment in rural Bangladesh. Int J Pediatr Otorhinolaryngol.

[2] (1997). Guidelines for Audiologic Screening.

[3] Cunningham M, Cox EO, Committee on Practice and Ambulatory Medicine and the Section on Otolaryngology and Bronchoesophagology (2003). Hearing assessment in infants and children: recommendations beyond neonatal screening. Pediatrics.

[4] Roush J (2008). Identification of hearing loss and middle ear dysfunction in preschool and school age children (American Academy of Audiology, Report and Position Statement). Semin Hear.

[5] Liao WH, Lien CF, Young ST (2010). The Hearing Scale Test for hearing screening of school-age children. Int J Pediatr Otorhinolaryngol.

[6] Snijders T, Bosker R (1999). The Worship and Principles of the Church of England: A Sermon, Preached at the Opening of Christ's Church, in Great-Barrington, on Christ-Mass Day, M.DCC.LXIV.

[7] Meinke DK, Dice N (2007). Comparison of audiometric screening criteria for the identification of noise-induced hearing loss in adolescents. Am J Audiol.

[8] Liao W, Young S, Tang S, Shiao A, Wang S, Lien C (2010). A novel method for quick hearing assessment of children.

[9] Masalski M, Kręcicki T (2013). Self-test web-based pure-tone audiometry: validity evaluation and measurement error analysis. J Med Internet Res.

[10] Liao WH, Young ST, Lien CF, Wang SJ (2011). An audiometer to monitor progressive hearing change in school-aged children. J Med Screen.

[11] Masalski M, Grysiński T, Kręcicki T (2018). Hearing tests based on biologically calibrated mobile devices: comparison with pure-tone audiometry. JMIR Mhealth Uhealth.

[12] Honeth L, Bexelius C, Eriksson M, Sandin S, Litton J, Rosenhall U, Nyrén O, Bagger-Sjöbäck D (2010). An internet-based hearing test for simple audiometry in nonclinical settings: preliminary validation and proof of principle. Otol Neurotol.

[13] Sandström J, Swanepoel DW, Carel Myburgh H, Laurent C (2016). Smartphone threshold audiometry in underserved primary health-care contexts. Int J Audiol.

[14] Renda L, Selçuk OT, Eyigör H, Osma U, Yılmaz MD (2016). Smartphone based audiometric test for confirming the level of hearing; is it useable in underserved areas. J Int Adv Otol.

[15] Masalski M, Kipiński L, Grysiński T, Kręcicki T (2016). Hearing tests on mobile devices: evaluation of the reference sound level by means of biological calibration. J Med Internet Res.

[16] Bright T, Pallawela D (2016). Validated smartphone-based apps for ear and hearing assessments: a review. JMIR Rehabil Assist Technol.

[17] Abu-Ghanem S, Handzel O, Ness L, Ben-Artzi-Blima M, Fait-Ghelbendorf K, Himmelfarb M (2016). Smartphone-based audiometric test for screening hearing loss in the elderly. Eur Arch Otorhinolaryngol.

[18] Swanepoel DW, Myburgh HC, Howe DM, Mahomed F, Eikelboom RH (2014). Smartphone hearing screening with integrated quality control and data management. Int J Audiol.

[19] Foulad A, Bui P, Djalilian H (2013). Automated audiometry using apple iOS-based application technology. Otolaryngol Head Neck Surg.

[20] Martínez-Pérez B, de la Torre-Díez I, López-Coronado M (2013). Mobile health applications for the most prevalent conditions by the World Health Organization: review and analysis. J Med Internet Res.

[21] Xing Y, Fu Z, Wu X, Chen J (2016). Evaluation of Apple iOS-based automated audiometry. https://www.icacommission.org/Proceedings/ICA2016BuenosAires/papers/ICA2016-0114.pdf.

[22] Masalski M, Grysiński T, Kręcicki T (2014). Biological calibration for web-based hearing tests: evaluation of the methods. J Med Internet Res.

[23] Ho CY, Li PC, Young ST (2017). Reference equivalent threshold sound pressure levels for Apple EarPods. J Acoust Soc Am.

[24] International Organization for Standardization.

[25] Levitt H (1971). Transformed up-down methods in psychoacoustics. J Acoust Soc Am.

[26] Flanary VA, Flanary CJ, Colombo J, Kloss D (1999). Mass hearing screening in kindergarten students. Int J Pediatr Otorhinolaryngol.

[27] Mason CA, Gaffney M, Green DR, Grosse SD (2008). Measures of follow-up in early hearing detection and intervention programs: a need for standardization. Am J Audiol.

[28] Khoza-Shangase K, Kassner L (2013). Automated screening audiometry in the digital age: exploring uHear™ and its use in a resource-stricken developing country. Int J Technol Assess Health Care.

[29] Cerwall P, Lundvall A, Jonsson P, Carson S, Möller R, Jonssonricsson MR (2018). GSMA.

[30] Bamford J, Fortnum H, Bristow K, Smith J, Vamvakas G, Davies L, Taylor R, Watkin P, Fonseca S, Davis A, Hind S (2007). Current practice, accuracy, effectiveness and cost-effectiveness of the school entry hearing screen. Health Technol Assess.

[31] Jimoh F, Lund EK, Harvey LJ, Frost C, Lay WJ, Roe MA, Berry R, Finglas PM (2018). Comparing diet and exercise monitoring using smartphone app and paper diary: a two-phase intervention study. JMIR Mhealth Uhealth.

[32] Swendeman D, Comulada WS, Koussa M, Worthman CM, Estrin D, Rotheram-Borus MJ, Ramanathan N (2018). Longitudinal validity and reliability of brief smartphone self-monitoring of diet, stress, and physical activity in a diverse sample of mothers. JMIR Mhealth Uhealth.

[33] El Shafie RA, Weber D, Bougatf N, Sprave T, Oetzel D, Huber PE, Debus J, Nicolay NH (2018). Supportive care in radiotherapy based on a mobile app: prospective multicenter survey. JMIR Mhealth Uhealth.

[34] Webb MJ, Wadley G, Sanci LA (2018). Experiences of general practitioners and practice support staff using a health and lifestyle screening app in primary health care: implementation case study. JMIR Mhealth Uhealth.

[35] Robbins RN, Gouse H, Brown HG, Ehlers A, Scott TM, Leu C, Remien RH, Mellins CA, Joska JA (2018). A mobile app to screen for neurocognitive impairment: preliminary validation of neuroscreen among HIV-infected South African adults. JMIR Mhealth Uhealth.

[36] March S, Day J, Zieschank K, Ireland M (2018). The Interactive Child Distress Screener: development and preliminary feasibility testing. JMIR Mhealth Uhealth.

[37] Paglialonga A, Tognola G, Pinciroli F (2015). Apps for hearing science and care. Am J Audiol.

[38] Yeh CH, Wei ST, Chen TW, Wang CY, Tsai MH, Lin CD (2014). A web-based audiometry database system. J Formos Med Assoc.

[39] Yimtae K, Israsena P, Thanawirattananit P, Seesutas S, Saibua S, Kasemsiri P, Noymai A, Soonrach T (2018). A tablet-based mobile hearing screening system for preschoolers: design and validation study. JMIR Mhealth Uhealth.

[40] Mahomed F, Swanepoel DW, Eikelboom RH, Soer M (2013). Validity of automated threshold audiometry: a systematic review and meta-analysis. Ear Hear.

[41] Margolis RH, Morgan DE (2008). Automated pure-tone audiometry: an analysis of capacity, need, and benefit. Am J Audiol.

[42] FitzZaland RE, Zink GD (1984). A comparative study of hearing screening procedures. Ear Hear.

[43] Sabo MP, Winston R, Macias JD (2000). Comparison of pure tone and transient otoacoustic emissions screening in a grade school population. Am J Otol.

[44] Gardner WG, Martin KD (1995). HRTF measurements of a KEMAR. J Acoust Soc Am.

